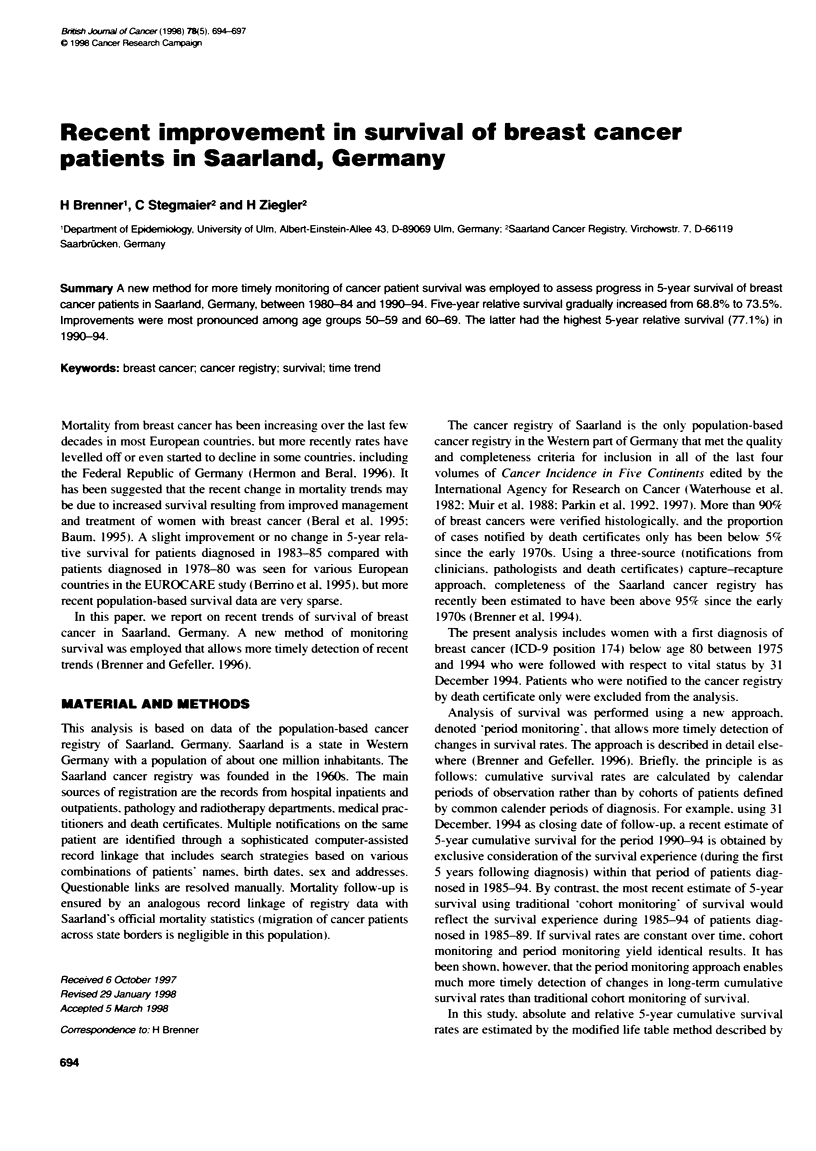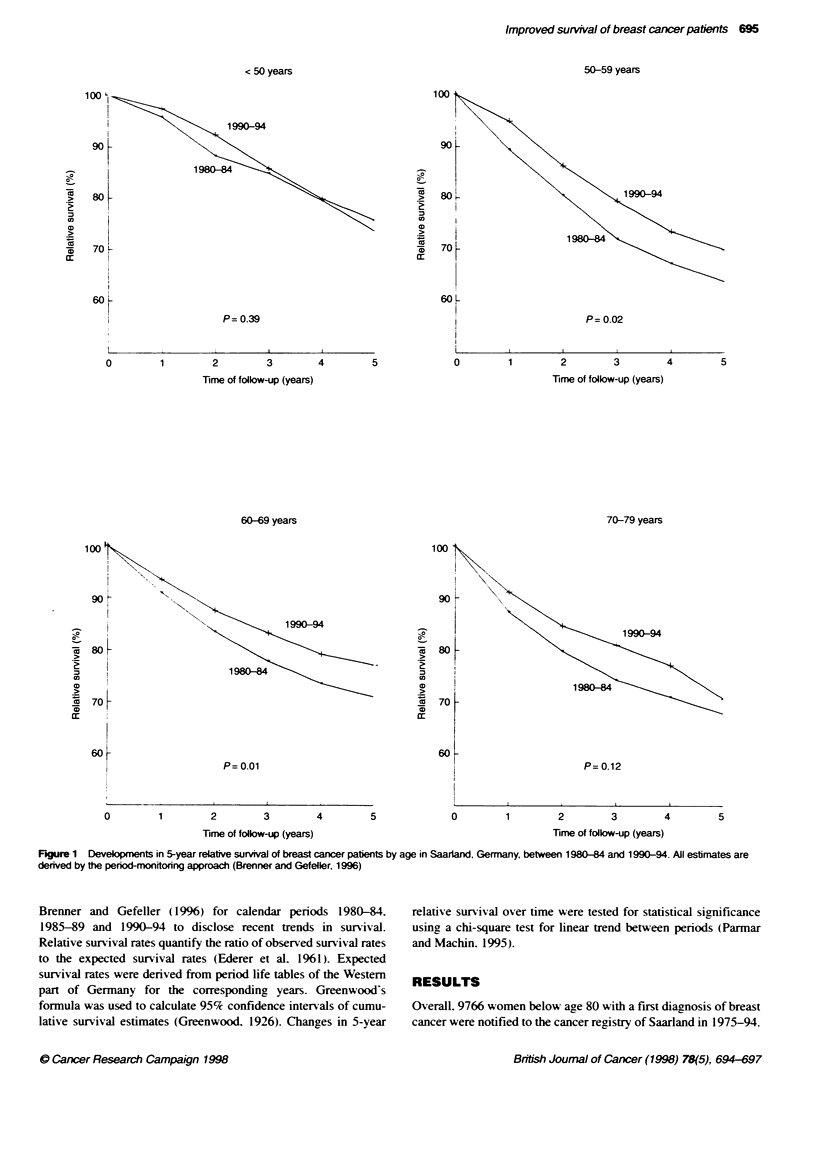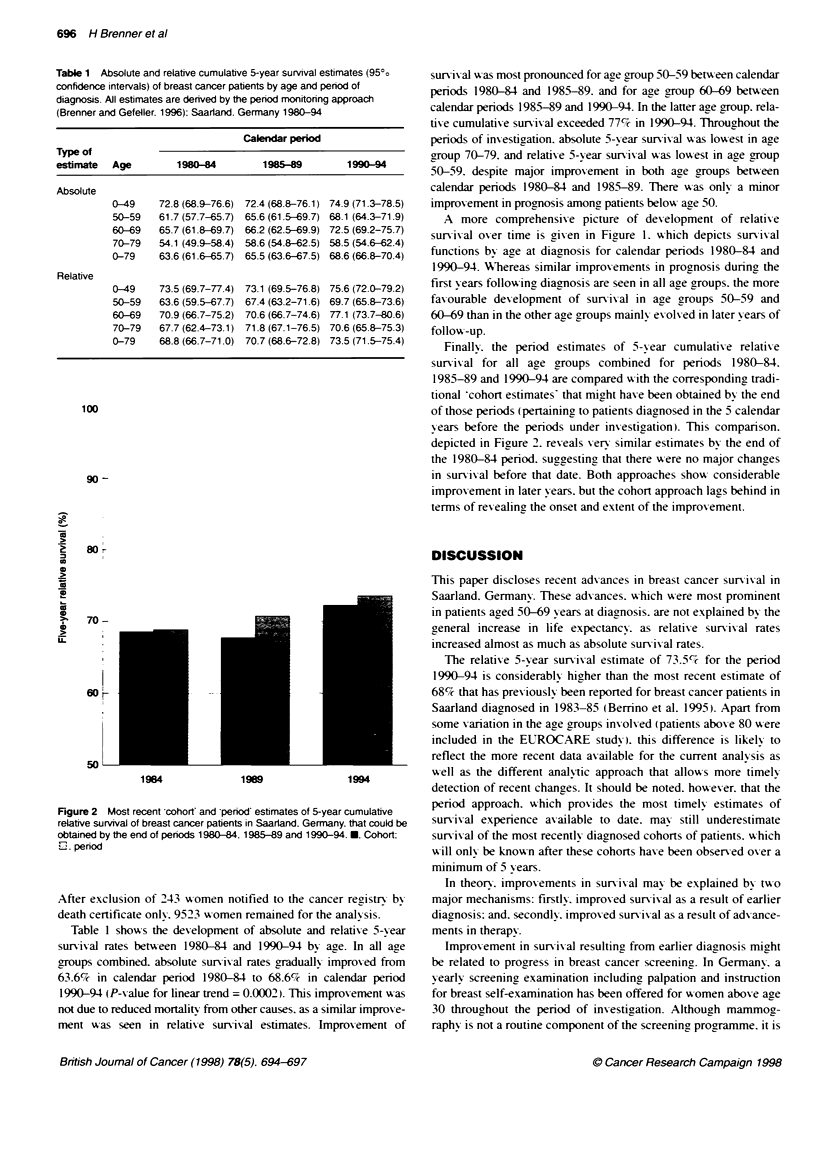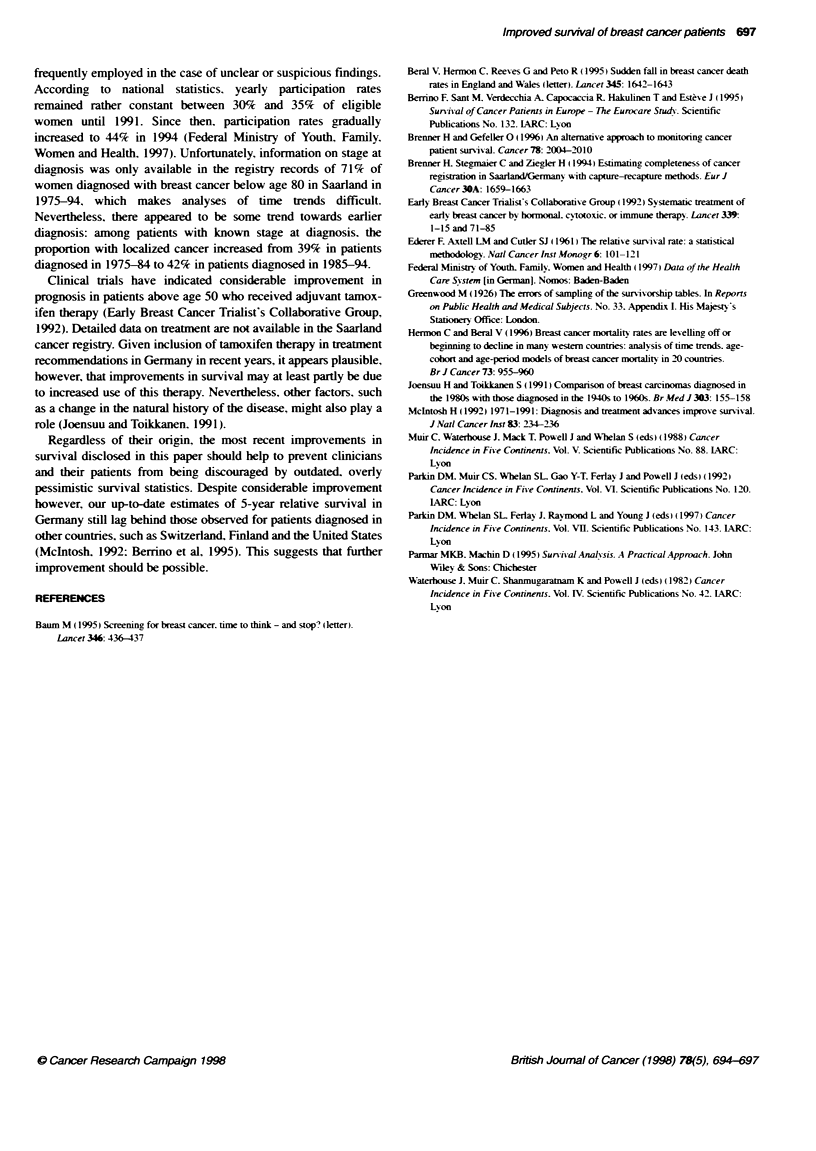# Recent improvement in survival of breast cancer patients in Saarland, Germany.

**DOI:** 10.1038/bjc.1998.562

**Published:** 1998-09

**Authors:** H. Brenner, C. Stegmaier, H. Ziegler

**Affiliations:** Department of Epidemiology, University of Ulm, Germany.

## Abstract

A new method for more timely monitoring of cancer patient survival was employed to assess progress in 5-year survival of breast cancer patients in Saarland, Germany, between 1980-84 and 1990-94. Five-year relative survival gradually increased from 68.8% to 73.5%. Improvements were most pronounced among age groups 50-59 and 60-69. The latter had the highest 5-year relative survival (77.1%) in 1990-94.


British Journal of Cancer (1998) 78(5), 694–697
© 1998 Cancer Research Campaign

### Recent improvement in survival of breast cancer patients in Saarland, Germany

**H Brenner[1], C Stegmaier[2] and H Ziegler[2]**

[1]Department of Epidemiology, University of Ulm, Albert-Einstein-Allee 43, D-89069 Ulm, Germany; [2]Saarland Cancer Registry, Virchowstr. 7, D-66119 Saarbrücken, Germany

**Summary** A new method for more timely monitoring of cancer patient survival was employed to assess progress in 5-year survival of breast cancer patients in Saarland, Germany, between 1980–84 and 1990–94. Five-year relative survival gradually increased from 68.8% to 73.5%. Improvements were most pronounced among age groups 50–59 and 60–69. The latter had the highest 5-year relative survival (77.1%) in 1990–94.

**Keywords:** breast cancer; cancer registry; survival; time trend

Mortality from breast cancer has been increasing over the last few decades in most European countries, but more recently rates have levelled off or even started to decline in some countries, including the Federal Republic of Germany (Hermon and Beral, 1996). It has been suggested that the recent change in mortality trends may be due to increased survival resulting from improved management and treatment of women with breast cancer (Beral et al, 1995; Baum, 1995). A slight improvement or no change in 5-year relative survival for patients diagnosed in 1983–85 compared with patients diagnosed in 1978–80 was seen for various European countries in the EUROCARE study (Berrino et al, 1995), but more recent population-based survival data are very sparse.

In this paper, we report on recent trends of survival of breast cancer in Saarland, Germany. A new method of monitoring survival was employed that allows more timely detection of recent trends (Brenner and Gefeller, 1996).

### MATERIAL AND METHODS

This analysis is based on data of the population-based cancer registry of Saarland, Germany. Saarland is a state in Western Germany with a population of about one million inhabitants. The Saarland cancer registry was founded in the 1960s. The main sources of registration are the records from hospital inpatients and outpatients, pathology and radiotherapy departments, medical practitioners and death certificates. Multiple notifications on the same patient are identified through a sophisticated computer-assisted record linkage that includes search strategies based on various combinations of patients' names, birth dates, sex and addresses. Questionable links are resolved manually. Mortality follow-up is ensured by an analogous record linkage of registry data with Saarland's official mortality statistics (migration of cancer patients across state borders is negligible in this population).

*Received 6 October 1997*
*Revised 29 January 1998*
*Accepted 5 March 1998*

*Correspondence to:* H Brenner

The cancer registry of Saarland is the only population-based cancer registry in the Western part of Germany that met the quality and completeness criteria for inclusion in all of the last four volumes of *Cancer Incidence in Five Continents* edited by the International Agency for Research on Cancer (Waterhouse et al, 1982; Muir et al, 1988; Parkin et al, 1992, 1997). More than 90% of breast cancers were verified histologically, and the proportion of cases notified by death certificates only has been below 5% since the early 1970s. Using a three-source (notifications from clinicians, pathologists and death certificates) capture–recapture approach, completeness of the Saarland cancer registry has recently been estimated to have been above 95% since the early 1970s (Brenner et al, 1994).

The present analysis includes women with a first diagnosis of breast cancer (ICD-9 position 174) below age 80 between 1975 and 1994 who were followed with respect to vital status by 31 December 1994. Patients who were notified to the cancer registry by death certificate only were excluded from the analysis.

Analysis of survival was performed using a new approach, denoted 'period monitoring', that allows more timely detection of changes in survival rates. The approach is described in detail elsewhere (Brenner and Gefeller, 1996). Briefly, the principle is as follows: cumulative survival rates are calculated by calendar periods of observation rather than by cohorts of patients defined by common calender periods of diagnosis. For example, using 31 December, 1994 as closing date of follow-up, a recent estimate of 5-year cumulative survival for the period 1990–94 is obtained by exclusive consideration of the survival experience (during the first 5 years following diagnosis) within that period of patients diagnosed in 1985–94. By contrast, the most recent estimate of 5-year survival using traditional 'cohort monitoring' of survival would reflect the survival experience during 1985–94 of patients diagnosed in 1985–89. If survival rates are constant over time, cohort monitoring and period monitoring yield identical results. It has been shown, however, that the period monitoring approach enables much more timely detection of changes in long-term cumulative survival rates than traditional cohort monitoring of survival.

In this study, absolute and relative 5-year cumulative survival rates are estimated by the modified life table method described by

**Figure 1** Developments in 5-year relative survival of breast cancer patients by age in Saarland, Germany, between 1980–84 and 1990–94. All estimates are derived by the period-monitoring approach (Brenner and Gefeller, 1996)

Brenner and Gefeller (1996) for calendar periods 1980–84, 1985–89 and 1990–94 to disclose recent trends in survival. Relative survival rates quantify the ratio of observed survival rates to the expected survival rates (Ederer et al, 1961). Expected survival rates were derived from period life tables of the Western part of Germany for the corresponding years. Greenwood's formula was used to calculate 95% confidence intervals of cumulative survival estimates (Greenwood, 1926). Changes in 5-year relative survival over time were tested for statistical significance using a chi-square test for linear trend between periods (Parmar and Machin, 1995).

### RESULTS

Overall, 9766 women below age 80 with a first diagnosis of breast cancer were notified to the cancer registry of Saarland in 1975–94.

**Table 1** Absolute and relative cumulative 5-year survival estimates (95% confidence intervals) of breast cancer patients by age and period of diagnosis. All estimates are derived by the period monitoring approach (Brenner and Gefeller. 1996): Saarland. Germany 1980–94

| Type of estimate | Age | Calendar period 1980–84 | 1985–89 | 1990–94 |
|---|---|---|---|---|
| Absolute | | | | |
| | 0–49 | 72.8 (68.9–76.6) | 72.4 (68.8–76.1) | 74.9 (71.3–78.5) |
| | 50–59 | 61.7 (57.7–65.7) | 65.6 (61.5–69.7) | 68.1 (64.3–71.9) |
| | 60–69 | 65.7 (61.8–69.7) | 66.2 (62.5–69.9) | 72.5 (69.2–75.7) |
| | 70–79 | 54.1 (49.9–58.4) | 58.6 (54.8–62.5) | 58.5 (54.6–62.4) |
| | 0–79 | 63.6 (61.6–65.7) | 65.5 (63.6–67.5) | 68.6 (66.8–70.4) |
| Relative | | | | |
| | 0–49 | 73.5 (69.7–77.4) | 73.1 (69.5–76.8) | 75.6 (72.0–79.2) |
| | 50–59 | 63.6 (59.5–67.7) | 67.4 (63.2–71.6) | 69.7 (65.8–73.6) |
| | 60–69 | 70.9 (66.7–75.2) | 70.6 (66.7–74.6) | 77.1 (73.7–80.6) |
| | 70–79 | 67.7 (62.4–73.1) | 71.8 (67.1–76.5) | 70.6 (65.8–75.3) |
| | 0–79 | 68.8 (66.7–71.0) | 70.7 (68.6–72.8) | 73.5 (71.5–75.4) |

**Figure 2** Most recent 'cohort' and 'period' estimates of 5-year cumulative relative survival of breast cancer patients in Saarland. Germany. that could be obtained by the end of periods 1980–84. 1985–89 and 1990–94. ■. Cohort: ◻. period

After exclusion of 243 women notified to the cancer registry by death certificate only. 9523 women remained for the analysis.

Table 1 shows the development of absolute and relative 5-year survival rates between 1980–84 and 1990–94 by age. In all age groups combined. absolute survival rates gradually improved from 63.6% in calendar period 1980–84 to 68.6% in calendar period 1990–94 ($P$-value for linear trend = 0.0002). This improvement was not due to reduced mortality from other causes. as a similar improvement was seen in relative survival estimates. Improvement of survival was most pronounced for age group 50–59 between calendar periods 1980–84 and 1985–89. and for age group 60–69 between calendar periods 1985–89 and 1990–94. In the latter age group. relative cumulative survival exceeded 77% in 1990–94. Throughout the periods of investigation. absolute 5-year survival was lowest in age group 70–79. and relative 5-year survival was lowest in age group 50–59. despite major improvement in both age groups between calendar periods 1980–84 and 1985–89. There was only a minor improvement in prognosis among patients below age 50.

A more comprehensive picture of development of relative survival over time is given in Figure 1. which depicts survival functions by age at diagnosis for calendar periods 1980–84 and 1990–94. Whereas similar improvements in prognosis during the first years following diagnosis are seen in all age groups. the more favourable development of survival in age groups 50–59 and 60–69 than in the other age groups mainly evolved in later years of follow-up.

Finally. the period estimates of 5-year cumulative relative survival for all age groups combined for periods 1980–84. 1985–89 and 1990–94 are compared with the corresponding traditional 'cohort estimates' that might have been obtained by the end of those periods (pertaining to patients diagnosed in the 5 calendar years before the periods under investigation). This comparison. depicted in Figure 2. reveals very similar estimates by the end of the 1980–84 period. suggesting that there were no major changes in survival before that date. Both approaches show considerable improvement in later years. but the cohort approach lags behind in terms of revealing the onset and extent of the improvement.

### DISCUSSION

This paper discloses recent advances in breast cancer survival in Saarland. Germany. These advances. which were most prominent in patients aged 50–69 years at diagnosis. are not explained by the general increase in life expectancy. as relative survival rates increased almost as much as absolute survival rates.

The relative 5-year survival estimate of 73.5% for the period 1990–94 is considerably higher than the most recent estimate of 68% that has previously been reported for breast cancer patients in Saarland diagnosed in 1983–85 (Berrino et al. 1995). Apart from some variation in the age groups involved (patients above 80 were included in the EUROCARE study). this difference is likely to reflect the more recent data available for the current analysis as well as the different analytic approach that allows more timely detection of recent changes. It should be noted. however. that the period approach. which provides the most timely estimates of survival experience available to date. may still underestimate survival of the most recently diagnosed cohorts of patients. which will only be known after these cohorts have been observed over a minimum of 5 years.

In theory. improvements in survival may be explained by two major mechanisms: firstly. improved survival as a result of earlier diagnosis: and. secondly. improved survival as a result of advancements in therapy.

Improvement in survival resulting from earlier diagnosis might be related to progress in breast cancer screening. In Germany. a yearly screening examination including palpation and instruction for breast self-examination has been offered for women above age 30 throughout the period of investigation. Although mammography is not a routine component of the screening programme. it is

© Cancer Research Campaign 1998

frequently employed in the case of unclear or suspicious findings. According to national statistics, yearly participation rates remained rather constant between 30% and 35% of eligible women until 1991. Since then, participation rates gradually increased to 44% in 1994 (Federal Ministry of Youth, Family, Women and Health, 1997). Unfortunately, information on stage at diagnosis was only available in the registry records of 71% of women diagnosed with breast cancer below age 80 in Saarland in 1975–94, which makes analyses of time trends difficult. Nevertheless, there appeared to be some trend towards earlier diagnosis: among patients with known stage at diagnosis, the proportion with localized cancer increased from 39% in patients diagnosed in 1975–84 to 42% in patients diagnosed in 1985–94.

Clinical trials have indicated considerable improvement in prognosis in patients above age 50 who received adjuvant tamoxifen therapy (Early Breast Cancer Trialist's Collaborative Group, 1992). Detailed data on treatment are not available in the Saarland cancer registry. Given inclusion of tamoxifen therapy in treatment recommendations in Germany in recent years, it appears plausible, however, that improvements in survival may at least partly be due to increased use of this therapy. Nevertheless, other factors, such as a change in the natural history of the disease, might also play a role (Joensuu and Toikkanen, 1991).

Regardless of their origin, the most recent improvements in survival disclosed in this paper should help to prevent clinicians and their patients from being discouraged by outdated, overly pessimistic survival statistics. Despite considerable improvement however, our up-to-date estimates of 5-year relative survival in Germany still lag behind those observed for patients diagnosed in other countries, such as Switzerland, Finland and the United States (McIntosh, 1992; Berrino et al, 1995). This suggests that further improvement should be possible.

### REFERENCES

Baum M (1995) Screening for breast cancer, time to think – and stop? (letter). *Lancet* **346**: 436–437

Beral V, Hermon C, Reeves G and Peto R (1995) Sudden fall in breast cancer death rates in England and Wales (letter). *Lancet* **345**: 1642–1643

Berrino F, Sant M, Verdecchia A, Capocaccia R, Hakulinen T and Estève J (1995) *Survival of Cancer Patients in Europe – The Eurocare Study*. Scientific Publications No. 132. IARC: Lyon

Brenner H and Gefeller O (1996) An alternative approach to monitoring cancer patient survival. *Cancer* **78**: 2004–2010

Brenner H, Stegmaier C and Ziegler H (1994) Estimating completeness of cancer registration in Saarland/Germany with capture–recapture methods. *Eur J Cancer* **30A**: 1659–1663

Early Breast Cancer Trialist's Collaborative Group (1992) Systematic treatment of early breast cancer by hormonal, cytotoxic, or immune therapy. *Lancet* **339**: 1–15 and 71–85

Ederer F, Axtell LM and Cutler SJ (1961) The relative survival rate: a statistical methodology. *Natl Cancer Inst Monogr* **6**: 101–121

Federal Ministry of Youth, Family, Women and Health (1997) *Data of the Health Care System* [in German]. Nomos: Baden-Baden

Greenwood M (1926) The errors of sampling of the survivorship tables. In *Reports on Public Health and Medical Subjects*. No. 33, Appendix I. His Majesty's Stationery Office: London.

Hermon C and Beral V (1996) Breast cancer mortality rates are levelling off or beginning to decline in many western countries: analysis of time trends, age-cohort and age-period models of breast cancer mortality in 20 countries. *Br J Cancer* **73**: 955–960

Joensuu H and Toikkanen S (1991) Comparison of breast carcinomas diagnosed in the 1980s with those diagnosed in the 1940s to 1960s. *Br Med J* **303**: 155–158

McIntosh H (1992) 1971–1991: Diagnosis and treatment advances improve survival. *J Natl Cancer Inst* **83**: 234–236

Muir C, Waterhouse J, Mack T, Powell J and Whelan S (eds) (1988) *Cancer Incidence in Five Continents*, Vol. V. Scientific Publications No. 88. IARC: Lyon

Parkin DM, Muir CS, Whelan SL, Gao Y-T, Ferlay J and Powell J (eds) (1992) *Cancer Incidence in Five Continents*, Vol. VI. Scientific Publications No. 120. IARC: Lyon

Parkin DM, Whelan SL, Ferlay J, Raymond L and Young J (eds) (1997) *Cancer Incidence in Five Continents*, Vol. VII. Scientific Publications No. 143. IARC: Lyon

Parmar MKB, Machin D (1995) *Survival Analysis. A Practical Approach*. John Wiley & Sons: Chichester

Waterhouse J, Muir C, Shanmugaratnam K and Powell J (eds) (1982) *Cancer Incidence in Five Continents*, Vol. IV. Scientific Publications No. 42. IARC: Lyon

© Cancer Research Campaign 1998